# Testosterone-Mediated Aggression in Angelman Syndrome Treated With Leuprolide and Orchiectomy

**DOI:** 10.7759/cureus.24865

**Published:** 2022-05-09

**Authors:** Danyon J Anderson, Mokshal H Porwal, Jay I Sandlow

**Affiliations:** 1 Urology, Medical College of Wisconsin, Wauwatosa, USA

**Keywords:** orchiectomy, leuprolide, hypersexuality, aggression, angelman syndrome

## Abstract

Angelman syndrome (AS) is a rare genetic imprinting disorder characterized by a maternal microdeletion of the 15q11q13 locus. It is traditionally associated with intellectual disability, inappropriate laughing, and a happy demeanor. Here, we report a patient with AS who presented with aggression and hypersexuality and was successfully treated with leuprolide injections for nine years until a definitive orchiectomy was performed. To the best of our knowledge, this is the first report of castration as a treatment for refractory behavioral symptoms in a patient with AS.

## Introduction

Patients with Angelman syndrome (AS) may exhibit behavioral manifestations including hyperactivity, aggression, and anxiety [1-3]. Aggression has been found to worsen with age; however, the underlying etiology of progression is unclear. Understanding the etiology of worsening aggression in AS patients could elucidate possible treatments for their aggression. Here, we present a patient with AS who displayed severe aggression and hypersexuality. He was treated with leuprolide injections for nine years before an orchiectomy was performed. To our knowledge, this is the first case report to describe pharmacologic castration and orchiectomy as a treatment for aggression in AS.

## Case presentation

A 16-year-old male with AS with a history of severe global delay presented with hypersexual stimulation, frustration, and aggressive behavior. Some of his atypical behaviors included grabbing and hitting his penis repeatedly. In addition, he was becoming more aggressive with his parents by scratching, pinching, and hitting repeatedly. His serum testosterone level on presentation was 550 ng/dL (normal: 300-1000 ng/dL).

His aggressive symptoms progressed for years and were not well controlled by behavioral therapy. In a visit to a urologist for evaluation of incontinence, an investigational leuprolide injection of 22.5 mg was offered for behavioral control. The ethics committee approved this trial. His mother noted a slight increase in adverse behavior for the first couple of weeks after his first injection after which his behavior remarkably improved. His mother was amazed by the improvement in his overall behavior since he was no longer grabbing, hitting, pinching, or scratching himself or others. She also stated that he focused better on variable tasks and diversions. However, three months later, she thought that he started to have a minor recurrence of some of the old behavior. At this time, a 30 mg injection of leuprolide was administered, and a follow-up was scheduled for four months. After three months, he rapidly started to become much more aggressive with scratching, clawing, and tearing things off the wall for several weeks. A repeat leuprolide injection of 30 mg was given with a follow-up scheduled in three months.

**Figure 1:**
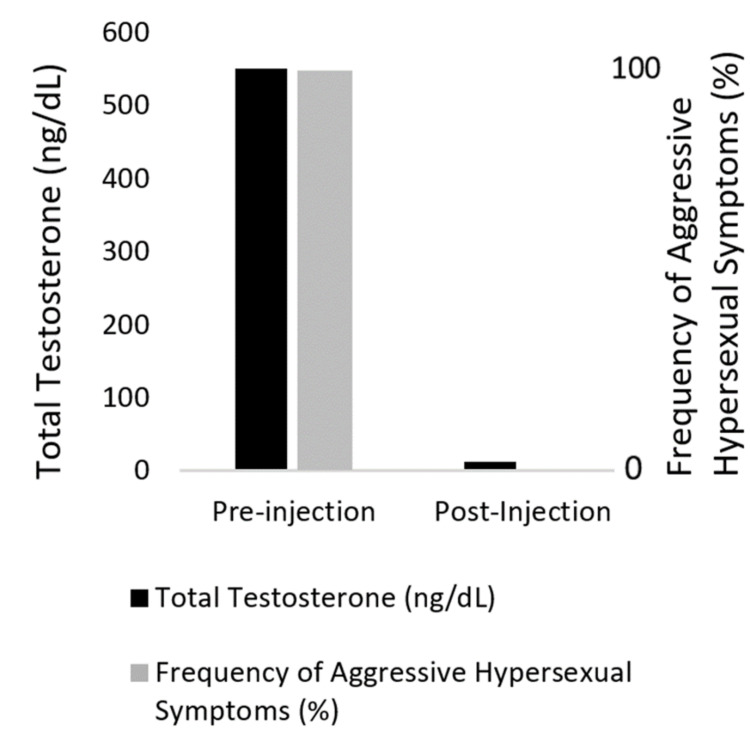
Pre- and post-injection total testosterone (ng/dL) and frequency of aggressive hypersexual symptoms (%). Pre-injection total testosterone was taken before the first leuprolide dose. Post-injection total testosterone was taken after leuprolide doses (n = 3) to verify efficacy. The presence or absence of aggressive hypersexual symptoms (n = 17) was defined by the patient’s mother’s desire for further treatment of excessive masturbation, biting, scratching, and hitting.

Leuprolide injections (30 mg) continued to be given every three months for four years. Testosterone was sporadically measured during this time and was found to be suppressed with post-injection serum testosterone levels of 4-20 ng/dL (Figure 1). Concomitant with decreased serum testosterone levels, the patient's parents noted significant improvements in the patient's behavior. They had previously expressed safety concerns caring for him. These three-month interval injections made his parents feel safe caring for him. Attempts to delay injection frequency to four months resulted in deterioration of behavior over the final two to four weeks before a dosage.

At this point, his mother stated that his aggression levels increased greatly. Due to difficulty extending the interval between injections as well as concerns regarding sequelae of long-term leuprolide use, injections were discontinued and replaced with off-label use of 5 mg finasteride daily.

Over the course of the next year, the patient’s mother reported that he was showing signs of increased acne and excessive masturbation. He was unable to fall asleep and stayed up until 1:00 am consistently. Additionally, the patient was back to pinching and hitting when his mother tried to divert his attention. Due to treatment failure with finasteride, leuprolide injections were resumed every three months per endocrinology recommendations; however, the dosage was reduced to 22.5 mg. Subsequently, the patient's aggressive behaviors were again ameliorated.

After two years of good behavior control on leuprolide, the patient's family could no longer proceed with leuprolide injections due to the lack of insurance coverage for this medication. In the few months following termination of treatment, the patient’s behavior was stable; however, in the following months, aggression, poor sleep, poor appetite, and hyperoral and sexual behaviors returned. The patient’s mother was having difficulties coping with and managing the patient’s behavior. She was opposed to giving her son sedative treatments; therefore, they considered orchiectomy as a permanent treatment. For the next two years, the patient’s family paid out-of-pocket for intermittent leuprolide injections. At this point, the patient’s family and care team decided to move forward with an orchiectomy as a permanent treatment. An ethics committee was consulted due to the controversial nature of removing testes as a treatment for hypersexuality and aggression. The ethics committee deemed it reasonable to pursue an orchiectomy given that having children may not be feasible due to the patient’s dependence on his parents for activities of daily living. Therefore, bilateral orchiectomy was successfully performed. Now 11 months post-procedure, the patient's mother reports that the patient has not been pinching, hitting, biting, or scratching himself or others and has not been masturbating inappropriately.

## Discussion

AS is typically associated with severe intellectual disability, speech impairments, hand-flapping movements, hyperactivity, seizures, and a happy demeanor with frequent laughing and smiling [4]. However, aggressive behaviors are common in people with AS. A behavioral study showed aggression in 10 of 12 children with AS and defined its purpose as an escape from undesirable situations [5]. Since people with AS often cannot use words to voice discontent, they may resort to aggression to communicate displeasure. Our patient was aggressive toward his parents when they tried to stop his hypersexual behaviors. When he exhibited less hypersexual behavior, parental stoppage of desired sexual activity decreased, which coincided with less aggression. Therefore, his aggression appeared to be a consequence of his hypersexuality and nonverbal status.

Our patient with AS exhibited hypersexuality and aggression. His symptoms decreased as his gonadotrophin and testosterone levels were depleted. The progression of aggression with age in AS may be associated with testosterone. Leuprolide controlled his symptoms. Eventually, orchiectomy was performed as a permanent treatment for his symptoms. Castration may be efficacious in the treatment of certain refractory and severe behavioral symptoms in some patients with AS. However, castration, even in patients unable to support children, raises significant ethical questions and considerations.

## Conclusions

Leuprolide and orchiectomy may be effective treatments in some AS patients with hypersexuality and associated aggression. However, this treatment may generate ethical concerns due to permanent infertility and changes in physical appearance. We endorse orchiectomy as an acceptable treatment in certain men with AS who have refractory hypersexuality and severe aggression. However, further studies and case reports exploring the relationship between aggression and testosterone in AS and the resolution of symptoms with castration would be needed first.
